# Back Volumes

**Published:** 1869-03-15

**Authors:** 


### *Back Volumes.*

A. W. Dewey, M. D., of Cicero, Hamilton County, Indiana, has some twelve or thirteen back volumes of THE JOURNAL which he writes us he is willing to dispose of.

